# Mind the Gap: Unraveling the Intricate Dance Between Alzheimer’s Disease and Related Dementias and Bone Health

**DOI:** 10.1007/s11914-023-00847-x

**Published:** 2024-01-29

**Authors:** Sonali J. Karnik, Tyler J. Margetts, Hannah S. Wang, Alexandru Movila, Adrian L. Oblak, Jill C. Fehrenbacher, Melissa A. Kacena, Lilian I. Plotkin

**Affiliations:** 1 Department of Orthopaedic Surgery, Indiana University School of Medicine, Indianapolis, IN 46202 USA; 2 Department of Biomedical Sciences and Comprehensive Care, Indiana University School of Dentistry, Indianapolis, IN 46202 USA; 3 Indiana Center for Musculoskeletal Health, Indiana University School of Medicine, Indianapolis, IN USA; 4 Department of Radiology & Imaging Sciences, Stark Neurosciences Research Institute, Indiana University School of Medicine, Indianapolis, IN 46202 USA; 5 Stark Neurosciences Research Institute, Indiana University School of Medicine, Indianapolis, IN 46202 USA; 6 Department of Pharmacology and Toxicology, Indiana University School of Medicine, Indianapolis, IN 46202 USA; 7 Richard L. Roudebush VA Medical Center, Indianapolis, IN 46202 USA; 8 Department of Anatomy, Cell Biology & Physiology, Indiana University School of Medicine, Indianapolis, IN 46202 USA

**Keywords:** Osteoporosis, Alzheimer’s disease, Fracture, Neuroinflammation, Inflammaging, Estrogen, AI, artificial intelligence, ChatGPT

## Abstract

**Purpose of Review:**

This review examines the linked pathophysiology of Alzheimer’s disease/related dementia (AD/ADRD) and bone disorders like osteoporosis. The emphasis is on “inflammaging”—a low-level inflammation common to both, and its implications in an aging population.

**Recent Findings:**

Aging intensifies both ADRD and bone deterioration. Notably, ADRD patients have a heightened fracture risk, impacting morbidity and mortality, though it is uncertain if fractures worsen ADRD. Therapeutically, agents targeting inflammation pathways, especially Nuclear factor kappa-light-chain-enhancer of activated B cells (NF-kB) and TNF-α, appear beneficial for both conditions. Additionally, treatments like Sirtuin 1 (SIRT-1), known for anti-inflammatory and neuroprotective properties, are gaining attention.

**Summary:**

The interconnectedness of AD/ADRD and bone health necessitates a unified treatment approach. By addressing shared mechanisms, we can potentially transform therapeutic strategies, enriching our understanding and refining care in our aging society. This review article is part of a series of multiple manuscripts designed to determine the utility of using artificial intelligence for writing scientific reviews.

## Introduction

This is one of many articles evaluating the assistance of using AI to write scientific review articles on musculoskeletal topics [1]. The first draft of this review was written by ChatGPT 4.0 but was edited and carefully checked for accuracy resulting in a final manuscript which was significantly different from the original draft. Refer to this edition’s Comment paper for more information [2]. Alzheimer’s disease and related dementias (ADRD) and osteoporosis represent two prominent health issues with global implications as their prevalence is soaring in tandem with an increasingly aging demographic. Historically perceived as independent ailments, there is mounting evidence of a complex entanglement between these diseases, signifying a nuanced interplay between neuronal and skeletal health [3–9]. ADRDs, which account for the largest proportion of dementia cases, are characterized by progressive cognitive decline. ADRDs presently affect approximately 53 million individuals globally, a number projected to triple by 2050 [10–12]. A notable feature of ADRD epidemiology is the significant gender bias, with women constituting nearly two-thirds of the diagnosed cases [13]. This discrepancy was thought to be due to women’s longer lifespan; however, latest research shows a more complex picture with hormonal interplay post-menopause, differences in brain structure and physiology in different sexes, socio-economic factors instead of longevity, and other factors yet to be well understood [14–18].

Concurrently, osteoporosis is a systemic skeletal condition characterized by diminished bone mass and compromised bone tissue microarchitecture. This alteration predisposes affected individuals to an increased fracture risk, rendering osteoporosis a substantial public health challenge [19–22]. Like ADRD, osteoporosis prevalence amplifies with age and is more prevalent in women, especially post-menopausal women, partially due to the discontinuation of protective estrogen levels [23–25]. Fractures, especially hip fractures, contribute to significant morbidity, functional decline, and heightened mortality, further complicating the clinical trajectory for those concurrently dealing with ADRD [26–29].

The interconnection of ADRD and bone health, which encompasses conditions such as osteoporosis and subsequent fractures, has sparked considerable scientific interest. Recent studies indicate a network of shared risk factors, including advanced age, gender, genetic susceptibility, and lifestyle factors, which all play a key role in mediating the pathophysiology of both ADRD and bone-related disorders [5, 8, 9, 13, 30, 31, 32•]. Additionally, the molecular hallmarks intrinsic to ADRD, such as chronicinflammation, oxidative stress, and the accumulation of amyloid-beta (Aβ) and tau proteins, have been linked with dysregulated bone metabolism and elevated fracture risk [33, 34, 35, 36, 37, 38, 39, 40, 41]. A study in experimental AD-like mouse model (APP/PS1) found Aβ to be expressed in bones and potentially involved in the pathogenesis of osteoporosis in this mouse model of AD [41]. Amyloid precursor protein (APP) and Aβ42 were found to be elevated in the osteoporotic tissues collected from bone biopsies of female patients with osteoporosis compared to healthy controls [4]. Mouse models of AD have shown low bone mineral density (BMD) and altered osteogenesis and osteoclastogenesis [42••, 43, 44, 45, 46, 47•]. Alternatively, impaired bone health may expedite AD progression, instigating a vicious cycle of disease advancement and deteriorating quality of life [5, 6, 30, 48]. Neuroinflammation within the brain is one of the hallmarks of ADRD and leads to progression of the and in turn, ADRD can increase the inflammatory response leading to a positive feedback loop resulting in the worsening of the condition [49]. Low-grade systemic inflammation due to aging or bone disorders can also lead to progression of ADRD [7, 38, 50]. Since inflammation is a common theme in ADRD, bone disorders, fracture healing, and aging; we discuss it in detail and in context of these linked and intertwined conditions in the following sections.

**Fig. 1:**
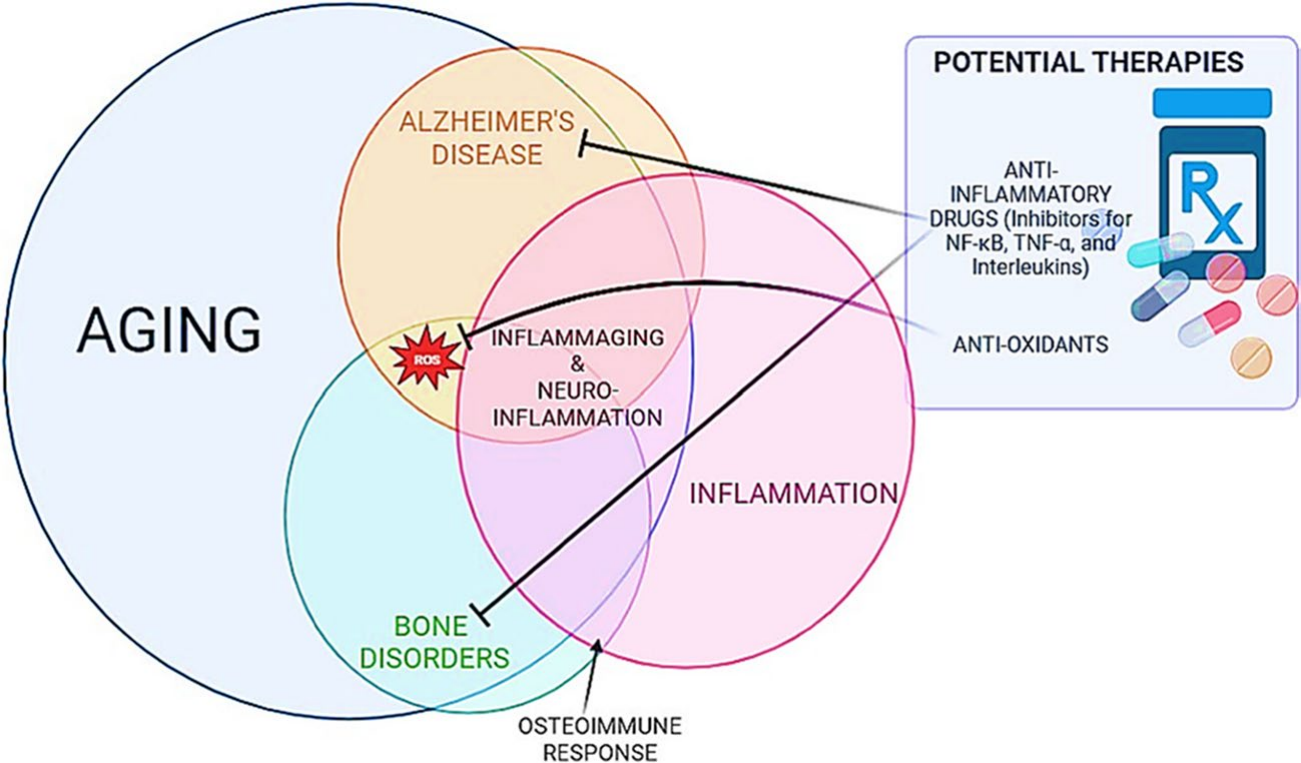
Overlapping factors between aging, inflammation, bone disorders, and ADRD. Potential therapies can be developed to target both ADRD and bone disorders such as osteoporosis that reduce inflammation and/or target ROS

This review aims to coalesce our present understanding of the reciprocal relationship between ADRD and poor bone health, delving into common risk factors, overlapping molecular pathways, and implications for fracture risk and osteoporosis (Fig. 1). Further, we highlight potential therapeutic strategies that might target both conditions simultaneously. Deciphering this intricate interface could significantly enhance clinical management and prognosis for millions of patients globally living with ADRD and bone disorders.

## Sexual Dimorphism in Alzheimer’s Disease Incidence: A Complex Confluence of Factors

The epidemiology of ADRD demonstrates a marked sex-based disparity, with women shouldering a higher burden of disease incidence compared to men [13]. In the USA, for instance, women constitute nearly two-thirds of the total ADRD patient population [13]. This disproportion cannot be wholly relegated to the well-documented longevity advantage women have over men, inviting a closer scrutiny of multifactorial contributors including hormonal dynamics, sex, genetics, and physiological differences [14, 17, 32, 51, 52].

Sex hormones, particularly estrogen, have been suggested to exert a crucial modulatory influence on ADRD risk [17, 53–57]. Estrogen exerts several neuroprotective effects, such as mitigating oxidative stress, stimulating neuronal growth, and fine-tuning synaptic plasticity [15, 57–61]. Estrogen is known to decrease inflammation in the brain [62]. Studies on the effect of estrogen on inflammatory factors show complex interactions and sometimes opposing effects between inflammation and estrogen [63–68]. In neuroinflammation, pro-inflammatory factors, such as cyclooxygenases 1 and 2 (COX1 and 2), are found to be elevated [69, 70]. Immune cells located in the brain (microglia and astrocytes) are also involved in increasing the expression of pro-inflammatory factors. COX 1 and 2 generate prostaglandins such as PGE_2_ [71]. Estrogen is known to have a protective effect by inhibiting or counteracting the inflammatory factors such as COX 1 and 2, prostaglandins, inducible nitric oxide, and aromatases [72–74].

Intriguingly, estrogen is also implicated in inhibiting the aggregation of beta-amyloid plaques, a defining neuropathological feature of AD, thereby potentially attenuating disease risk [54, 60]. Menopause results in a pronounced reduction in estrogen levels in women, which may, in turn, amplify their susceptibility to ADRD [16]. A study in female 3xTg mice (a transgenic mouse model for AD) demonstrated the roles female sex hormones play in AD pathology [55]. In 2007, Carroll et al. [55] showed that administering estrogen to ovariectomized female 3xTg mice had beneficial effects, reducing Aβ accumulation and improving working memory-based performance in a Y-maze test [55]. Progesterone on the other hand blocked estrogen’s beneficial action on Aβ accumulation, but not cognitive performance, when administered together with estrogen. Progesterone, however, was beneficial in tau pathology by reducing phosphorylation of tau, when either administered alone or in combination with estrogen [55]. This study highlights the importance and synergistic action of female sex hormones, estrogen and progesterone, in 3xTg mice. However, menopausal women do not completely lack ovaries and the secretion of sex hormones is only reduced and not eliminated as is the case in ovariectomized female mice. Hence, caution needs to be exercised in interpreting the results in animal models and translating them to humans. Another study involving 3xTg mice showed that blocking follicle stimulating hormone (FSH), by FSH binding antibody (FSH-Ab), slowed the progression of AD in 3xTg mice [75••]. FSH blockade resulted in a reduction in Aβ and Tau levels in ovariectomized 3xTg mice. The study also showed that FSH acts on the hippocampal and cortical neurons to accelerate Aβ accumulation. Studies done in another AD mouse model, 5xFAD, also shows beneficial effects of estrogen [57] and membrane-associated estrogen receptor, G protein-coupled receptor 30 (GPR30) [76]. FSH has also been explored as a potential target for different diseases including AD and osteoporosis. It was demonstrated by Gera et al. that blocking FSH in mice using a humanized antibody against FSH helped stimulate new bone formation [77, 78]. More studies are needed in different female mouse models for AD that study the effects of estrogen, progesterone, FSH, and luteinizing hormone (LH) synergistically. These nuances will likely impact the translatability of animal studies to humans, especially when considering potential therapies.

Emerging evidence also points toward a potential role of the female sex chromosome gene expression in shaping ADRD risk [31]. Certain genetic variants, such as the apolipoprotein E (APOE) ε4 allele, a well-documented genetic risk factor for AD, appear to confer a heightened risk in women compared to men [79]. Moreover, physiological differences between sexes, encompassing differences in brain structure and functionality, may contribute to sex-dependent incidence of ADRD. Women are reported to exhibit a higher prevalence of brain alterations such as pronounced atrophy in regions prone to ADRD, potentially augmenting disease risk [80]. However, whether atrophy in the regions of brain is a cause or an effect of ADRD is yet to be fully understood. Furthermore, the cause of brain atrophy and its relationship to sex is not fully understood. Due to the critical role that APOE isoforms play in ADRD pathology, mice expressing human APOE (h-APOE) isoforms have been developed and used for studying ADRD pathology [81]. These mice show human isoform-specific differences in lipid physiology and synaptic function which are hallmarks of ADRD pathogenesis [81], suggesting that the allele ε4 in human APOE shows highest genetic risk for ADRD compounded by female sex [79, 82].

A comprehensive understanding of sex-based differences in ADRD incidence could offer critical insights into the pathophysiological underpinnings of the disease, possibly informing the development of sex-specific therapeutic strategies. Further research is warranted to decode the mechanistic basis of this gender disparity in ADRD, as well as potential intersections with bone health and fracture risk.

## Convergence of Central Nervous System and Peripheral Inflammation: Roles in ADRD and Bone Health

The physiological process of aging is also accompanied by “inflammaging,” a chronic, low-grade inflammatory state believed to be a significant contributor to both neurodegenerative and skeletal diseases [83]. This inflammatory response in aging, ADRD, and osteoporosis shares common molecular pathways. These include the activation of the Nuclear factor kappa-light-chain-enhancer of activated B cells (NF-kB) signaling pathway, an increase in the production of reactive oxygen species (ROS), and the emergence of cellular senescence [84–86].

In 2016, DiSabato et al. [87] defined neuroinflammation as an inflammatory response in the brain and spinal cord, mediated by inflammatory cytokines, chemokines, and ROS. The intricate pathogenesis of ADRD prominently features an augmented state of neuroinflammation, a phenomenon increasingly acknowledged as a crucial accelerator of the disease trajectory [88–92]. This persistent inflammation within the brain is primarily orchestrated by microglia and astrocytes, the resident immune cells and support cells of the central nervous system (CNS), respectively, which exhibit a heightened levels of activity within an ADRD environment [93–97].

In a physiologically normal state, microglia enact an essential role in preserving brain homeostasis. They function as innate immune cells, facilitating the clearance of cellular debris and aberrant proteins, inclusive of Aβ peptides [98–100]. However, as ADRD advances, these microglial cells undergo a significant phenotypic transformation, instigating a potent neuroinflammatory response marked by the production of pro-inflammatory cytokines, ROS, and additional neurotoxic entities such as Aβ and tau tangles [89, 94, 95, 98]. This phenotypic switch in microglial behavior is further exacerbated by the progressive accumulation of AD hallmark structural pathological features, namely Aβ plaques and neurofibrillary tangles composed of hyperphosphorylated tau proteins [101].

Astrocytes, another cell type implicated in the genesis of neuroinflammation, contribute to the inflammatory cascade within the ADRD-afflicted brain [96, 97, 102, 103]. Under pathological circumstances, these cells transition into a hyperactive state, upregulating the expression of inflammatory mediators while simultaneously forfeiting their capacity to support neuronal health and synaptic functionality [104–106].

The neuroinflammatory response not only augments neurodegeneration but also appears to underpin other pathological facets of ADRD, such as synaptic dysfunction and disruption of neuronal networks [87, 107, 108]. Moreover, mounting evidence proposes that neuroinflammation may arise early in the ADRD continuum, potentially even preceding the formation of Aβ plaques and tau tangles. This places neuroinflammation squarely in the crosshairs as a viable therapeutic target [109–111].

Persistent neuroinflammation in ADRD implies a plausible link to altered bone metabolism and heightened fracture risk, though the precise molecular mediators bridging these processes remain to be unraveled [3, 4, 6, 9, 30, 48, 112]. While neuroinflammation primarily affects the central nervous system (CNS), it can have systemic effects as well. Studies have shown that neuroinflammation can communicate with the peripheral immune system, leading to the release of cytokines and other immune mediators into the bloodstream. This process is known as the “neuroimmune interface.” The communication between the CNS and the peripheral immune system is bidirectional. Peripheral cytokines can cross the blood-brain barrier and enter the brain, contributing to neuroinflammation. Conversely, pro-inflammatory molecules released by activated immune cells within the brain can signal to peripheral immune cells, triggering an inflammatory response in the periphery. Therefore, in the context of brain neuroinflammation, it is possible to observe an increase in peripheral cytokines. The specific cytokines released can vary depending on the underlying neuroinflammatory condition. For example, in conditions like multiple sclerosis or stroke, the peripheral levels of cytokines such as interleukin-6 (IL-6), tumor necrosis factor-alpha (TNF-α), and interleukin-1 beta (IL-1β) have been found to be elevated.

It is important to note that the relationship between brain neuroinflammation and peripheral cytokines is complex and can be influenced by various factors. Additionally, the exact mechanisms and consequences of this bidirectional communication are still being studied. As we refine our understanding of neuroinflammation’s role in ADRD, it becomes imperative to further scrutinize its association with bone health. Such investigations may uncover novel therapeutic strategies, shedding new light on our approach to this formidable disease.

The importance of inflammation is equally apparent in the context of bone health. Pro-inflammatory cytokines, such as TNF-α, IL-1β, and IL-6, have been implicated in osteoclastogenesis, the formation of bone resorbing osteoclasts [113, 114]. Upregulation of osteoclastogenesis can precipitate bone loss and subsequent osteoporosis [115, 116]. Additionally, chronic inflammation can thwart bone formation by inhibiting the function and differentiation of bone forming osteoblasts, thereby tipping the balance of bone homeostasis towards resorption [117, 118].

Emerging evidence suggests a potential bidirectional interplay between ADRD and bone health, with inflammation acting as a critical mediator. Neuroinflammation in ADRD may indirectly influence bone health via dysregulation of the hypothalamic-pituitary-adrenal (HPA) axis, which could lead to elevated cortisol production and, consequently, bone loss [119–121]. Conversely, systemic inflammation ensuing from osteoporosis and fractures could augment neuroinflammation and further propagate ADRD pathology [7, 39, 86, 122–124].

Observational studies in humans following surgeries showed an increase in cognitive decline and is called postoperative cognitive dysfunction (POCD) [125–128]. Several studies have suggested that abdominal and cardiac surgery can induce neuroinflammatory responses [125, 129, 130]. For example, research in animal models has shown that surgical trauma can result in increased levels of pro-inflammatory cytokines within the brain [129]. Additionally, clinical studies have found evidence of neuroinflammatory markers in the cerebrospinal fluid of patients undergoing abdominal and cardiothoracic surgeries [131–133]. It is worth noting that the extent and duration of neuroinflammation following abdominal or cardiothoracic surgery may vary among individuals and depend on factors such as the type of surgery, the individual’s overall health, and the presence of pre-existing conditions. The consequences of neuroinflammation after surgery are still being studied, and more research is needed to fully understand its implications.

Therefore, targeting the shared inflammatory pathways could hold therapeutic potential for both ADRD and bone disorders. Unraveling these intertwined pathways could reveal innovative therapeutic targets and strategies that concurrently address ADRD and compromised bone health, offering a more holistic and effective approach to improving patient prognosis.

## Alzheimer’s Disease and Fractures: A Connection Worthy of Investigation

In the current section, we will explore the link between ADRD and the propensity for fractures in ADRD patients. We will also explore the potential link between fractures leading to ADRD progression and cognitive decline. Understanding the connection between fractures and cognitive decline is imperative as this connection is not well understood and very few studies exist to date.

Emerging evidence suggests a compelling link between ADRD and increased susceptibility to fractures, a common manifestation of deteriorating bone health. Fractures, particularly in the elderly, can significantly compromise quality of life and escalate morbidity and mortality rates, underlining the necessity for further exploration of this relationship [134–136].

Clinical studies indicate that individuals with ADRD are at a substantially higher risk of sustaining fractures, primarily attributable to increased falls and decreased BMD [3, 134, 137, 138]. Indeed, altered lifestyle factors associated with ADRD, including decreased physical activity and poor nutrition, can lead to compromised BMD and overall poor bone health [8, 138], further increasing the propensity for fractures [29, 139]. Additionally, cognitive impairment in ADRD can lead to a higher risk of falls and subsequent fractures due to improper gait [29].

One of the intriguing aspects of this relationship is whether fractures can contribute to the progression of ADRD. Even though there are no animal studies that suggest that systemic inflammation resulting from a fracture could exacerbate pre-existing ADRD pathology, several clinical observational studies suggest that there might be an association between fractures and ADRD progression [140, 141•]. However, human studies present a more complex picture. While no direct association between fracture and the rate of cognitive decline in ADRD patients has been established, fractures can indirectly impact cognitive and functional outcomes in these patients, possibly due to complications such as immobility, pain, and the use of sedative medications post-fracture [29, 142]. As discussed earlier, surgeries can lead to POCD with neuroinflammation playing a main role [125–128]. Hence, it would not be surprising to see similarities in human patients and cognitive decline following fracture surgeries. Strategies to minimize fracture risk, such as fall prevention measures and maintaining bone health through adequate nutrition and exercise, become crucial components of patient management [143–146].

In summary, a complex interplay exists between ADRD and fractures. While individuals with ADRD appear more susceptible to fractures, the potential impact of fractures on ADRD progression is yet to be definitively established. Given the significant implications of these findings, further comprehensive studies are warranted to fully understand this bidirectional link, which could potentially uncover novel avenues for patient management and therapeutic interventions.

## Potential Therapeutic Interventions for Alzheimer’s Disease: A Focus on Neuroinflammation and Overlapping Pathways with Bone Health Disorders

Emerging evidence highlighting the intersection between ADRD and bone health—specifically through shared inflammatory pathways—provides new opportunities for the development of therapeutic strategies that could concurrently address both conditions [6–9]. Given the pivotal role that inflammation plays in both ADRD and osteoporosis, anti-inflammatory agents are a key focus of current research. However, due to the complex nature and intertwined pathways shared between ADRD and bone health, the approaches taken should not be limited only to anti-inflammatory agents but should also include bioactive molecules targeting multiple pathways.

Non-steroidal anti-inflammatory drugs (NSAIDs), conventionally used for pain and inflammation management, have been examined for their potential role in ADRD progression. Though research has produced mixed results, some epidemiological studies involving long-term NSAID usage suggest a decreased ADRD incidence, thereby indicating potential therapeutic benefits [147–150]. The putative mechanism of action is the mitigation of inflammation, thereby decelerating the disease progression. Correspondingly, NSAIDs could potentially offer protection against bone loss by inhibiting prostaglandin synthesis, a process known to stimulate osteoclast activity and subsequent bone resorption [151, 152].

Another therapeutic target of interest is the NF-kB signaling pathway, a central regulator of inflammation and immune responses, implicated in both ADRD and osteoporosis [153, 154]. Drugs inhibiting NF-kB have demonstrated promising results in preclinical models by reducing neuroinflammation and improving cognitive function in ADRD, and by attenuating bone loss in osteoporosis [155–158]. Denosumab is a human monoclonal antibody that binds to receptor activator of NF-κB ligand (RANKL) approved by the FDA for the treatment of post-menopausal osteoporosis [159, 160]. RANKL is indispensable for osteoclast (OC) differentiation and activation [160]. OCs resorb bone and an increase in OCs leads to higher bone resorption and osteoporosis. Denosumab, by blocking RANKL, inhibits OC differentiation and activation. RANKL has also been found to be expressed in normal rodent brain [161]; however, its role in neuronal functions and neuroprotection is not completely understood. Given the intermingled pathways involved in both bone health and disorders as well as ADRD progression [7], Denosumab as a potential candidate for treating neuroinflammation and osteoporosis is intriguing and might need to be explored as a potential dual target osteoporosis and ADRD therapy.

Romosozumab, a humanized monoclonal antibody used to treat osteoporosis, inhibits sclerostin, a molecule expressed by osteocytes and involved with inhibition of osteogenesis via Wnt/β-catenin pathway [162, 163]. In a study conducted to compare the performance of Denosumab and Romosozumab in treating osteoporotic patients, the authors observed a significant increase in BMD of the lumbar spine, total hip, and femoral neck after 12 months of starting the treatments [164]. Wnt signaling is also involved in synaptic plasticity and amyloid pathology in AD [165]. Activation of the Wnt signaling pathway has also been involved in microglial survival [166]. Intracerebrovascular injections of sclerostin in ICR mice reduced the dendritic complexity of pyramidal neurons in the hippocampus showing that there might be an overlap of pathways such as Wnt signaling via sclerostin and neuronal dendritic growth [167]. Since inhibition of sclerostin would activate Wnt signaling, and this might benefit bone formation as well as potentially benefit neurons, synaptic plasticity, and microglia, Romosozumab might also be a good candidate for both osteoporosis and ADRD therapy.

Biological therapies, including monoclonal antibodies that target pro-inflammatory cytokines (for example, TNF-α and IL-1β), may also offer potential benefits for ADRD and bone disorders [168–175]. Preliminary studies in rodents suggest these therapies could reduce neuroinflammation and slow cognitive decline in ADRD, while concurrently inhibiting OC activity and reducing bone loss in osteoporosis [168–171].

Despite the potential of the monoclonal antibodies as dual target treatments for both osteoporosis and ADRD, what needs to be kept in mind is that under normal conditions, monoclonal antibodies do not cross the blood brain barrier (BBB) and therefore would not be able to neutralize locally produced proteins of interest in the brain [176]. To make these biological molecules a viable option to treat ADRD, these need to be delivered in a way that can cross the BBB [176, 177]. However, recently there have been some studies that show that modifying large molecules such as monoclonal antibodies as an IgG fusion may help deliver them to the target proteins past the BBB [178]. The BBB transport system involves two modalities to deliver biological molecules, namely receptor-mediated transcytosis (RMT) for large molecules and carrier-mediated transport (CMT) for small molecules. Exploiting the biology and chemistry of these endogenous modalities of BBB transport can lead to effective and efficient delivery of monoclonal antibodies and other drugs across BBB, making dual target treatment for ADRD and osteoporosis a reality in the future [179].

Emerging research has underscored the potential therapeutic role of hormones, such as estrogen, which have shown protective effects on both brain and bone health. Estrogen replacement therapy following menopause may reduce ADRD risk and slow disease progression, while concurrently preventing postmenopausal bone loss [180–183]. FSH is also involved in regulating bone mass and has been described as a key hormone, along with estrogen, that modifies the risk of developing osteoporosis in post-menopausal women [184]. As mentioned earlier, blocking FSH in the 3xTg AD mouse model slowed the progression of AD [75••]. The findings from AD mouse models for FSH blockade as well as beneficial effect of FSH blockade on bone mass show a promise for exploring FSH either as a standalone therapy for targeting both osteoporosis and ADRD or in combination with estrogen replacement therapy in post-menopausal women.

Similarly, calcitonin gene-related peptide (CGRP) has shown promise as a target in preclinical models for its neuroprotective effects and for its traditional application in treating osteoporosis [185–187]. The flip side to CGRP treatment is that peripheral CGRP is also involved in the pathophysiology of migraine. CGRP levels were elevated in the plasma, cerebrospinal fluid, and tear samples from migraine patients [188–190] and intravenous administration of CGRP to migraineurs can trigger headache [191]. Currently, there are FDA-approved drugs used to treat migraine that target either CGRP or its receptor in patients [192]. This is the major clinical conundrum that needs to be considered while exploring CGRP or CGRP receptor as potential targets for treating osteoporosis and ADRD.

One family of molecules, Sirtuins, has been studied and explored for their involvement in aging and longevity. Sirtuins are a class of deacetylases that are NAD+ dependent [193–196]. Among the seven Sirtuins, Sirtuin 1 (SIRT1) has been most extensively studied for its role as an anti-inflammatory molecule; in addition, it protects against ROS, is anti-apoptotic, and is involved in energy metabolism in brain and bone health [195, 197–203]. SRT1720 (a SIRT1 activator) is known to enhance endothelial cell function and promote angiogenesis in 20–22-month mice which may be beneficial for fracture healing [204]. Since SIRT1 not only acts on one axis, such as inflammation or ROS, for its protective role, it may be a good candidate for developing therapies for ADRD as well as bone disorders. Even though there are numerous studies on mouse models of AD and SIRT1 as a neuroprotective agent [205–211] and the role of SIRT1 in preventing bone loss [203, 212, 213], there are no studies to date that explored the effect of SIRT1 as having a potential dual protection in reducing AD while preventing bone loss.

However, as promising as these therapeutic strategies may be, it is crucial to evaluate the potential side effects and risks associated with long-term use. Continued research is needed to substantiate these therapies’ effectiveness and safety through well-designed, large-scale clinical trials. Harnessing the shared pathological mechanisms between ADRD and bone health may potentially unlock innovative and integrated treatment approaches that could significantly improve the quality of life for those dealing with ADRD and/or osteoporosis.

## Conclusion

In conclusion, the intricate relationship between ADRD, bone disorders such as osteoporosis, and inflammation has emerged as a promising research frontier. An increased susceptibility to fractures, largely due to falls and reduced bone density, is observed in individuals with ADRD, potentially exacerbating morbidity and mortality in these patients. Interestingly, many studies on mouse models indicate that osteoporosis or low bone turnover states lead to exacerbation of hallmarks of ADRD. Therefore, it would not be surprising to find that fractures might also potentially intensify ADRD progression based on clinical follow-up studies on POCD after other types of surgeries [214]. As shown in Fig. 1, the overlap between aging, inflammation, ADRD, and osteoporosis is significant. Through this lens, we may uncover novel therapeutic targets, leading to integrated care strategies aimed at reducing fracture risk in ADRD patients and limiting the potential impact of fractures on ADRD progression. As we unravel the shared pathophysiological pathways in ADRD, bone health, and inflammation, we are not merely enriching our academic knowledge but taking crucial strides towards improving patient care in our aging population.
